# Effect of fecal microbiota transplantation on primary hypertension and the underlying mechanism of gut microbiome restoration: protocol of a randomized, blinded, placebo-controlled study

**DOI:** 10.1186/s13063-022-06086-2

**Published:** 2022-02-24

**Authors:** Luyun Fan, Jie Ren, Youren Chen, Yang Wang, Zihong Guo, Peili Bu, Jinfeng Yang, Wenjun Ma, Bingpo Zhu, Yanyan Zhao, Jun Cai

**Affiliations:** 1 Hypertension Center, State Key Laboratory of Cardiovascular Disease, National Center for Cardiovascular Diseases, Fuwai Hospital, Chinese Academy of Medical Sciences and Peking Union Medical College, Beijing, China; 2 Shanxi Bethune Hospital, Taiyuan, Shanxi China; 3 The Second Affiliated Hospital of Shantou University, Shantou, Guangdong China; 4 Medical Research & Biometrics Center, National Center for Cardiovascular Diseases, Fuwai Hospital Chinese Academy of Medical Sciences, Beijing, China; 5 Fuwai Yunnan Cardiovascular Hospital, Kunming, Yunnan China; 6 Qilu Hospital of Shandong University, Jinan, Shandong China; 7 The People’s Hospital of Ji Xian District, Tianjin, China; 8 Southern University of Science and Technology Hospital, Shenzhen, China

**Keywords:** Hypertension, Blood pressure, Fecal microbiota transplantation, Therapy, Randomized controlled trial, Microbiome, Metabolome

## Abstract

**Background:**

Hypertension is currently the leading modifiable cause of global morbidity and mortality, leading to substantial health and financial burdens. Although multiple studies of management models and innovative therapeutic strategies for hypertension have been conducted, there are still gaps in the field, with a poor control rate reflecting a lack of novel, effective, clinically translated medication or intervention options. Recent animal and human studies repeatedly confirmed a link between the microbiota and hypertension. Of note is our previous study establishing a cause-and-effect relationship between the gut microbiota and blood pressure elevation. A hypothesis of gut microbiota intervention for treating hypertension is thus postulated, and fecal microbiota transplantation (FMT) from healthy donors was performed.

**Methods:**

A multicenter, randomized, placebo-controlled, blinded clinical trial will be performed in 120 grade 1 hypertensive patients for 3 months. All recruited patients will be randomly assigned in a 1:1 ratio to take oral FMT capsules or placebo capsules on day 1, day 7, and day 14 and will be followed up on day 30, day 60, and day 90. The primary outcome is the change in office systolic blood pressure from baseline to day 30. The main secondary outcomes are BP indicators, including changes in systolic and diastolic blood pressure from office and 24-h ambulatory blood pressure monitoring; assessments of ankle-branchial index and pulse wave velocity; profiling of fecal microbial composition and function; profiling of fecal and serum metabolome; changes in levels of blood glucose, blood lipids, and body mass index; and assessment of adverse events as a measure of safety.

**Discussion:**

Expanding upon our previous research on the role of the gut microbiota in the pathogenesis of hypertension, this study serves as a clinical translation advancement and explores the potential of fecal microbiota transplantation for treating hypertension. The underlying mechanisms, particularly the roles of specific microorganisms or their postbiotics in blood pressure amelioration, will also be investigated via multiple approaches, such as metagenomic sequencing and metabolomic profiling.

**Trial registration:**

ClinicalTrials.govNCT04406129. Registered on May 28, 2020

## Administrative information

Note: The numbers in curly brackets in this protocol refer to SPIRIT Checklist item numbers. The order of the items has been modified to group similar items (see http://www.equator-network.org/reporting-guidelines/spirit-2013-statement-defining-standard-protocol-items-for-clinical-trials/).

**Table:** 

Title {1}	Effect of fecal microbiota transplantation on primary hypertension and the underlying mechanism of gut microbiome restoration: protocol of a randomized, blinded, placebo-controlled study
Trial registration {2a and 2b}.	ClinicalTrials.gov Identifier: NCT04406129 registered on May 28th, 2020, https://clinicaltrials.gov/ct2/show/NCT04406129.
Protocol version {3}	Version 3.3, dated 26 February 2021
Funding {4}	This work is funded by grants from the National Natural Science Foundation of China (Project ID. 81825002), the CAMS Innovation Fund for Medical Sciences (CIFMS, 2021-I2M-1-007), and from the Beijing Outstanding Young Scientist Program (Project ID. BJJWZYJH01201910023029).
Author details {5a}	1. Luyun Fan, M.D., Ph.D., Hypertension Center, State Key Laboratory of Cardiovascular Disease, National Center for Cardiovascular Diseases, Fuwai Hospital, Chinese Academy of Medical Sciences and Peking Union Medical College, Beijing, China. 2. Jie Ren, M.D., Shanxi Bethune Hospital, Taiyuan, Shanxi, China. 3. Youren Chen, M.D., The Second Affiliated Hospital of Shantou University, Shantou, Guangdong, China. 4. Yang Wang, Medical Research & Biometrics Center, National Center for Cardiovascular Diseases, Fuwai Hospital Chinese Academy of Medical Sciences, Beijing, China. 5. Zihong Guo, M.D., Fuwai Yunnan Cardiovascular Hospital, Kunming, Yunnan, China. 6. Peili Bu, M.D., Ph.D., Qilu Hospital of Shandong University, Jinan, Shandong, China. 7. Jinfeng Yang, M.D., The People’s Hospital of Ji Xian District, Tianjin, China. 8. Wenjun Ma, M.D., Ph.D., Hypertension Center, State Key Laboratory of Cardiovascular Disease, National Center for Cardiovascular Diseases, Fuwai Hospital, Chinese Academy of Medical Sciences and Peking Union Medical College, Beijing, China. 9. Bingpo Zhu, M.D., Southern University of Science and Technology Hospital, Shenzhen, China. 10. Yanyan Zhao, Medical Research & Biometrics Center, National Center for Cardiovascular Diseases, Fuwai Hospital Chinese Academy of Medical Sciences, Beijing, China. 11. Jun Cai, M.D., Ph.D., Hypertension Center, State Key Laboratory of Cardiovascular Disease, National Center for Cardiovascular Diseases, Fuwai Hospital, Chinese Academy of Medical Sciences and Peking Union Medical College, Beijing, China.
Name and contact information for the trial sponsor {5b}	Prof. Jun Cai (Principal Investigator), State Key Laboratory of Cardiovascular Disease, National Center for Cardiovascular Diseases, Fuwai Hospital, Chinese Academy of Medical Sciences and Peking Union Medical College; 167 Beilishi Road, Xichen District, 100037, Beijing, China; E-mail: caijun7879@126.com; caijun@fuwaihospital.org; Phone: 01088322161. Contact for public and scientific queries: Luyun Fan, E-mail: fuwai_fanluyun@163.com; fanluyun@fuwai.com.
Role of sponsor {5c}	The sponsor is the principal investigator of the study who procured funding. The study funders will not be involved in the study design, implementation, data interpretation, or result publication.

## Introduction

### Background and rationale {6a}

Cardiovascular diseases (CVDs) are currently the leading cause of global health loss, accounting for one-third of deaths worldwide [1], and it is estimated that CVDs will cause 7.8 million premature deaths in 2025 [2]. Primary hypertension is the most prevalent cardiovascular disease affecting ~ 30% of the adult population worldwide and has become a severe public health issue because of its low control rate, high morbidity rate, and involvement in ~ 19% of global deaths [3, 4]. Despite explorations on pharmacological and device-based therapies over the last 60 years, few treatment options are currently available, which stresses the importance of advances of novel hypotheses in this field.

Although blood pressure (BP) is regulated by multifactorial genetic and environmental factors, an increasing number of studies have indicated the role of the microbiota in BP homeostasis [5]. In germ-free (GF) animal models, angiotensin II (Ang II)-induced hypertension cannot be replicated [6]. Decreased diversity and discordant composition of the gut microbiota (compared to that of healthy controls) have been found in several hypertensive cohorts [7–10]. Fecal microbiota transplantation from multiple hypertensive animal models can increase BP in controls [11]. Our previous study first confirmed that transplants of hypertensive patient feces reshaped the gut microbiota of healthy GF mice and resulted in significant BP elevation [9]. Of note, modulating intestinal flora via transplantation from healthy rats could dramatically reduce BP in salt-induced hypertensive animals [12]. Oral antibiotics such as minocycline and vancomycin can assist in the treatment of hypertension [13]. Supplementary probiotics also modestly ameliorated high BP in rodents and/or humans [14–17]. A hypothesis is indeed raised that gut microbiota intervention might be a potential approach to ameliorate hypertension. Fecal microbiota transplantation (FMT) is a strategy for entire gut microbiome transplantation from healthy donors to the diseased recipient. Since 2013, FMT is recommended for treatment on recurrent Clostridium difficile infection resistant to standard-of-care therapies [18]. Potential therapeutic roles of FMT via affecting patients’ gut microbiota [19] has been examined on other gastrointestinal diseases such as inflammatory bowel disease(IBD) [20, 21] and irritable bowel disease [22], and extraintestinal diseases such as metabolic syndrome [23, 24], obese and type 2 diabetes mellitus [25, 26]. Of note, limited study of the entire gut microbial ecology restoration among hypertensive patients has been conducted, and the durability after FMT intervention remains unknown.

We herein developed a multicenter, randomized, placebo-controlled, blinded study utilizing an FMT intervention to explore the efficacy of gut microbiota intervention for the treatment of essential hypertension. We postulate that FMT intervention presents a better BP-lowering effect than placebo. Secondary outcomes also included changes in recipient fecal microbial composition and function, fecal and serum metabolome, and fasting blood glucose and lipid levels; ankle-branchial index (ABI); body mass index (BMI); and occurrence of adverse events as a measure of safety outcomes. 

### Objectives {7}

This study primarily aims to assess whether FMT ameliorates elevated blood pressure in patients with grade one essential hypertension. Additionally, this study will determine whether any BP changes are associated with alterations in the fecal microbiome and fecal and serum metabolomes of hypertensive patients when comparing these parameters measured prior to and after FMT. Of note are the microbial structure and functional alterations in the microbiota of the recipient and their durability. The durability of the clinical response after the initial response to FMT will also be evaluated in participants with hypertension. Other indicators, including fasting blood glucose and lipid levels, ABI, BMI, and occurrence of adverse events as a measure of safety outcomes, will also be assessed.

### Trial design {8}

This study is an investigator-initiated, multicenter, randomized, blinded, placebo-controlled clinical trial with a 3-month duration.

Assuming a true SBP difference between the “FMT capsule group” and “placebo group” with a mean of 5 mmHg and a standard deviation of 8.6 mmHg for SBP change per arm, a sample size of 96 grade one hypertensive patients yields > 80% statistical power to demonstrate a > 5 mmHg difference between arms at a 1-sided alpha level of 0.025. Given premature withdrawal or failure potentials before the primary end point data were collected, a 20% rate of loss-of-follow-up was calculated, resulting in the recruitment target of 120 participants with grade one hypertension who met the eligibility criteria. The participants will be randomized 1:1 to receive FMT via capsule or placebo capsules. The experimental group will then be offered three separate “FMT capsule” treatments (day 1, day 7, day 14), and the control group will then be offered three separate “placebo capsules” (day 1, day 7, day 14). In addition to the comprehensive examination before the intervention period and at the end of the trial, participants in both arms will be followed up at day 7, day 14, day 30, day 60, and day 90 to undergo an assessment of their clinical BP and other clinical indicators. Adverse events and treatment for other accompanying diseases will also be recorded at each visit, and fecal and blood samples will be collected before the trial and at each visit for future metagenomic and metabolomic profiling analysis. All staff involved in the clinical assessments, statistical analysis, and/or randomization of participants will be blinded.

The protocol report refers to the SPIRIT reporting guidelines [27].

## Methods: participants, interventions, and outcomes

### Study setting {9}

This study was conducted by Fuwai Hospital, Chinese Academy of Medical Sciences, National Center for Cardiovascular Diseases, State Key Laboratory of Cardiovascular Diseases. Patients with grade one essential hypertension will be recruited via outpatient clinics at implementation centers, including Fuwai Hospital, Chinese Academy of Medical Sciences (Beijing), Fuwai Yunnan Cardiovascular Hospital (Yunnan Province), Qilu Hospital of Shandong University (Shandong Province), the Second Affiliated Hospital of Shantou University Medical College (Guangdong Province), The People’s Hospital of Ji Xian District (Tianjin), Shanxi Bethune Hospital (Shanxi Province), and South University of Science and Technology Hospital (Shenzhen).

### Eligibility criteria {10}

The following are the inclusion criteria:

1. Age 18~60 years
2. Established diagnosis of grade one hypertension (initial diagnosis or no use of antihypertensive drugs within a month): 140 mmHg ≤ office SBP < 160 mmHg and/or 90 ≤ office DBP < 100 mmHg from three measurements on different days without any antihypertensive medications, according to the “2010 Chinese Guidelines for Prevention and Treatment of Hypertension” [28].
3. Participants who provide written informed consent after receiving a thorough explanation.

The following are the exclusion criteria:

1. Antibiotic or probiotic usage within one month before the study
2. Participation in other clinical trials related to hypertension currently or within the last 3 months
3. Antihypertensive medication usage currently or within the last month
4. Diagnosis of secondary hypertension
5. Severe hepatic or renal diseases (over 3-fold elevation of alanine transaminase, elevated serum creatinine over 2.5 mg/dl [221 μmol/L], estimated glomerular filtration rate less than 30 mL/min/1.73 m^2^, or end-stage kidney dysfunction requiring dialysis)
6. History of stroke, including hemorrhagic stroke and large artery atherosclerotic cerebral infarction (LAACI) but not transient ischemic attack (TIA) or lacunar infarction
7. History of hospitalization within the last 6 months due to myocardial infarction, history of coronary revascularization including percutaneous transluminal coronary intervention (PCI) or coronary artery bypass grafting (CABG) within the last year, anticipated PCI or CABG surgery in the following 12 months
8. Sustained atrial fibrillation or arrhythmias at recruitment disturbing the electronic BP measurement
9. NYHA class III–IV heart failure, hospitalization for chronic heart failure exacerbation within the last 6 months
10. Severe valvular diseases: potential for surgery or percutaneous valve replacement within the study period
11. Dilated cardiomyopathy; hypertrophic cardiomyopathy, rheumatic cardiac disease, congenital cardiac disease
12. Other severe diseases influencing the entry or survival of participants, such as malignant tumors or acquired immune deficiency syndrome
13. Cognitive impairment or severe neuropsychiatric comorbidities, making individuals incapable of providing their own informed consent
14. Participants preparing for or currently experiencing pregnancy and/or lactation
15. Other conditions deeming individuals inappropriate for recruitment according to the investigators

### Who will take informed consent? {26a}

Informed consent forms have been prepared according to the guidelines of and approved by the local ethical boards of all participating centers. The investigator or his or her designee will present the study to potential study participants and will answer study-related questions for the participants, such as study objectives, interventions, potential benefits, risks, and alternatives. The participants will read the informed consent forms and have the opportunity to communicate with their relatives prior to signing and dating the consent form. Participation in this study is voluntary and entirely up to the participants. Even if the participants do not participate, their medical services will not be affected in any way. If participants decide to participate, they also have the right to withdraw at any time and will not be influenced now or in the future regarding treatment. Once the participants decide to stop participating in the study, they will no longer be provided with the study interventions.

### Additional consent provisions for collection and use of participant data and biological specimens {26b}

Information on clinical data and fecal and blood sample collection is included in the informed consent form. The volume of fecal samples (5 g per visit) and blood samples (10 ml per visit) taken will not lead to side effects. Participation for each patient is confidential, and their identity will be confined within the study centers under participants’ agreement. To reserve options for validation and meta-analysis with future studies of FMT on hypertension, we will make our data available to other study groups after reasonable application. Coded samples may be shared with academic collaborators, including metagenomic profiles, metabolomic profiles, and clinical metadata. The sharing will be confirmed with the standard language of the informed consent, and all identifiable information will be excluded. Although current plans on potential collaboration are lacking, the options for sharing are reserved.

### Interventions

#### Explanation for the choice of comparators {6b}

According to the “2010 Chinese Guidelines for Prevention and Treatment of Hypertension”, lifestyle intervention is recommended as the first-line therapy for grade one hypertension treatment, and pharmacological interventions can be initiated after 3 months of lifestyle intervention and persistent hypertension. All participants in this study will be educated regarding lifestyle intervention and randomized to the “FMT capsule” intervention or the “placebo capsule” intervention. Participants in the “placebo capsule” group will take placebo capsules orally on day 1, day 7 (± 1 day), and day 14 (± 1 day). The placebo capsules have identically appearing powder that does not contain donor stool or any active drug.

#### Intervention description {11a}

Participants in the “FMT capsule” group will take the FMT capsules orally on day 1, day 7 (± 1 day), and day 14 (± 1 day). The FMT capsules (China National Intellectual Property Administration, patent ID. CN 104922158 B) are prepared by processing extensively screened donor stool in reference to the current consensus on FMT [19, 29–31] and stored at − 80 °C in freezers before recovery for use. Light, high-liquid meals on the day before intervention and fasting for at least 4 h prior to and 2 h after intervention are required.

#### Criteria for discontinuing or modifying allocated interventions {11b}

The discontinuation criteria include withdrawal of informed consent, loss to follow-up, occurrence of serious adverse events, complications, certain physiological changes or other medical reasons precluding continuation of the study according to the investigators’ assessment, and significant deviation from the intervention algorithm, which would probably affect the outcome evaluation of the study.

#### Strategies to improve adherence to interventions {11c}

Before inclusion, the willingness to comply will be repeatedly confirmed among potential participants. An advantage in regard to compliance is that all capsules are taken at three intervention visits within two weeks. The FMT capsules and placebo capsules will be stored, delivered, and recovered by investigators and/or patient care providers from the implementation centers. All capsules will be stored in − 80 °C freezers and delivered from the trial center into implementation centers via dry ice. Before the intervention, the investigators and/or patient care providers will recover capsules in a 37 °C water bath and make detailed records of the number of distributed, administered, and returned capsules. All recovered capsules at each visit will be orally taken within 30 min under clinical supervision. The remaining study capsules will be returned to the responsible center. The participant compliance rate will be evaluated as the actual number of capsules used divided by the anticipated number of capsules used × 100%. Good compliance is defined as a compliance rate of 80–120%; poor compliance is defined as a compliance rate less than 80% or more than 120%. If poor compliance is identified, thorough communication between investigators and patients will be performed to reach possible solutions. In addition, an assessment of the gut microbial composition between FMT capsules and stool samples from recipients at different time points will be performed to assess the engraftment of microbiota, which will provide additional information regarding participant adherence in part.

#### Relevant concomitant care permitted or prohibited during the trial {11d}

In reference to the hypertension guidelines, lifestyle modifications are recommended for all hypertensive patients and for all participants recruited in this study. During the treatment and follow-up period, participants will be asked to maintain a routine pattern of daily activities, such as physical activity, lifestyle, and habits. To minimize the interference caused by dietary environmental factors on the intestinal flora, recruited participants in each arm will be required to record their dietary history before each study visit and to make no dietary habit changes throughout the trial. Antihypertension medications and the use of probiotics, prebiotics, or antibiotics will be avoided during the study period except for in specific clinical conditions (i.e., persistent BP elevation over 180/110 mmHg) requiring pharmacological interventions on the basis of the physician’s evaluation. The conventional treatment for accompanying diseases in participants can remain unchanged and will be recorded. Treatment for adverse events is permitted and will be recorded.

#### Provisions for post-trial care {30}

In accordance with the informed consent form, appropriate medical care and assistance will be provided rather than direct financial compensation for participants who suffer harm relevant to trial participation. Additionally, close monitoring and management will be performed for those participants according to relevant laws and regulations.

### Outcomes {12}

Our primary hypothesis is a BP-lowering effect of gut microbiota restoration via FMT compared to the effects observed in the placebo group in grade one hypertensive patients. Accordingly, the primary outcome is defined as the change in office systolic blood pressure (SBP) level from baseline to the one-month follow-up (day 30).

Other indicators related to BP evaluation, safety concerns, and potential mechanism exploration will be assessed as the main secondary outcomes and include the following:

1. Change in office systolic blood pressure (SBP) level via office BP monitoring from baseline to day 7, day 14, day 60, and day 90 visits
2. Change in office diastolic blood pressure (DBP) level via office BP monitoring from baseline to day 7, day 14, day 30, day 60, and day 90 visits
3. Change in average SBP and DBP during the 24-h assessment, day, and night via 24-h ambulatory BP monitoring (ABPM) from baseline to day 30 and day 90 visits
4. Target organ damage assessment from baseline to the final visit (day 90 visit), including (a) the change in ankle-brachial blood pressure index (ABI) as an objective measurement of arterial insufficiency based on the ratio of ankle systolic pressure to brachial systolic pressure and (b) the change in pulse wave velocity as the distance divided by the time for a pulse delay between two arterial sites
5. Number of participants with adverse events (AEs) documented CRF as a measure of safety at baseline, day 7, day 14, day 30, day 60, and day 90 visits
6. Changes in fecal microbiota composition and function pre- and postintervention (FMT or placebo) via metagenomic analysis, stratified by (a) randomization and (b) change in office SBP level from baseline to day 7, day 14, day 30, day 60, and day 90 visits
7. Durability of engraftment of donor microbiome following FMT to day 7, day 14, day 30, day 60, and day 90 visits, measured by the similarity of fecal microbiota composition between donor and recipient
8. Changes in fecal metabolite composition and function pre- and postintervention (FMT or placebo) via metabolomic analysis, stratified by (a) randomization and (b) change in office SBP from baseline to day 7, day 14, day 30, day 60, and day 90 visits
9. Changes in blood metabolite composition and function pre- and postintervention (FMT or placebo) via metabolomic analysis, stratified by (a) randomization and (b) change in office SBP level from baseline to day 7, day 14, day 30, day 60, and day 90 visits
10. Change in fasting blood glucose levels from baseline to the final visit (day 90 visit)
11. Change in blood lipid levels (total cholesterol, total triglycerides, low-density lipoprotein cholesterol, and high-density lipoprotein cholesterol) from baseline to the final visit (day 90 visit)
12. Change in body mass index from baseline to the final visit (day 90 visit)

#### Participant timeline {13}

The participant timeline is presented in Table 1.

**Table 1:** The schedule of enrollment, interventions, and assessments

Workflow	***Enrollment***	***Allocation***	***Follow-up***					***Close-out***
	***− 7 days***	***Day 0***	***Day 1***	***Day 7***	***Day 14***	***Day 30***	***Day 60***	***Day 90***
**Enrollment**								
***Eligibility screening***	✓							
***Informed consent***	✓							
***Allocation***		✓						
**Interventions**								
***FMT capsules***			✓	✓	✓			
***Placebo capsules***			✓	✓	✓			
**Assessments**								
***Comorbid diseases and treatment***	✓							
***Physical examination***	✓							✓
***Office BP and heart rate***	✓			✓	✓	✓	✓	✓
***24-h ABPM***	✓					✓		✓
***Blood routine***	✓							✓
***Urine routine***	✓							✓
***Blood chemical test***	✓							✓
***Urine microalbumin, creatinine***	✓							✓
***ECG***	✓							✓
***Arterial stiffness (ABI, PWV)***	✓							✓
***Diet records***			✓	✓	✓	✓	✓	✓
***Patient compliance***			✓	✓	✓			
***Adverse events***			✓	✓	✓	✓	✓	✓
***Combined medications for accompanied diseases***			✓	✓	✓	✓	✓	✓
***Fecal sample collection***	✓			✓	✓	✓	✓	✓
***Blood sample collection***	✓			✓	✓	✓	✓	✓

Blood biochemical test: *ALT* alanine aminotransferase, *AST* aspartate aminotransferase, *K* potassium, *Na* sodium, *Cl* chloride, *BUN* urea nitrogen, *CREA* creatinine, *UA* uric acid, *TC* total cholesterol, *TG* triglyceride, *HDL-C* high-density lipoprotein cholesterol, *LDL-C* low-density lipoprotein cholesterol, *GLU* fasting blood glucose

#### Sample size {14}

Sample size calculation was performed via PASS version 15 software before trial initiation based on the following: (1) no relevant current data for the effect of FMT on hypertension; (2) a clinically significant improvement of a decrease in office SBP at least 5 mmHg associated with reduction in stroke (14%), cardiovascular disease (9%), and mortality (7%) [32]; (3) meta-analysis of the effectiveness of probiotics on hypertension based on randomized controlled clinical trials that revealed a reduction of SBP of 3.56 mmHg (95% CI, 6.46 to 0.66) [33]. Assuming a true SBP difference between the “FMT capsule group” and “placebo group” with a mean of 5 mmHg and a standard deviation of 8.6 mmHg for SBP change per arm, a sample size of 96 grade one hypertensive patients yields > 80% statistical power to demonstrate a > 5 mmHg difference between arms at a 1-sided alpha level of 0.025. Given the potential for premature withdrawal or failure before the primary end point data were collected, a 20% rate of loss-of-follow-up was considered, resulting in 120 participants with grade one hypertension according to the “2010 Chinese Guidelines for Prevention and Treatment of Hypertension” [28]. Participants were recruited and randomized 1:1 to receive FMT by capsule or placebo capsules.

#### Recruitment {15}

Eligible participants will be screened at the hypertension outpatient clinic of the implementation centers according to the eligibility criteria for the trial. Advertisements for the study will utilize onsite posters and multiple media platforms.

### Assignment of interventions: allocation

#### Sequence generation {16a}

Randomization allocation sequencing was performed at a 1:1 ratio in blocks of four and stratified by center, by an independent statistician via the SAS 9.4 software (SAS Institute, Cary NC, USA) and incorporated into a central computerized randomization system that was developed and managed by the Medical Research & Biometrics Center, National Center for Cardiovascular Diseases, Fuwai Hospital Chinese Academy of Medical Sciences.

#### Concealment mechanism {16b}

Only the staff from the Medical Research & Biometrics Center who will generate the random codes and an independent staff member from the responsible center (Fuwai Hospital) will maintain the overall random codes that will be incorporated into the Electronic Data Capture System (EDC system) and will maintain the sealed envelopes for each participant.

#### Implementation {16c}

Patient care providers and investigators from implementation centers will log in to the EDC system, enter the system login page, select the center, fill in the password, and enter the randomization information page. After inputting the name of the implementation center, qualified screening code, initials of participants’ name, age, sex, and other relevant information; passing the system check for information correction; and clicking the “submit for confirmation” button, the randomization date, qualified random number; and allocated arm of participants will be obtained. At this time, participants are enrolled in this study.

The eligible participants will be randomly divided into two arms: the “FMT capsule” group and the “placebo capsule” group which will receive FMT capsules or placebo capsules, respectively, three times in the outpatient department on day 1, day 7, and day 14 after the trial initiation. The random numbers are labeled on the package for capsules in accordance with the GCP. The first administration (day 1) will be commenced after randomization with medication compliance/adherence, adverse events, and treatment for comorbid diseases assessed and recorded via investigators and/or patient care providers.

### Assignment of interventions: blinding

#### Who will be blinded {17a}

Study participants and all personnel, including investigators, patient care providers, and/or data analysts who recruit and follow-up participants and collect and analyze data, will be blinded. The packaged intervention capsules and sealed emergency envelopes for each participant will be prepared by independent staff from the responsible center not involved in the study. The sealed envelopes are maintained at the responsible center for disclosure of arm allocation when necessary.

#### Procedure for unblinding if needed {17b}

If medical emergency events occur, unblinding can be performed if necessary via the responsible investigation team under the supervision of the principal investigator. The reason, date, location of unblinding, and signatures of investigators will be recorded in detail. All participants will be followed up until the close-out visits unless the withdrawal criteria are met.

### Data collection and management

#### Plans for assessment and collection of outcomes {18a}

All outcomes will be evaluated at seven time points, including baseline, the first intervention day, and 7 days, 14 days, 30 days, 60 days, and 90 days after intervention initiation. In addition to the kick-off meeting on February 26, 2021, all staff involved in the study were trained on a detailed study protocol, participant recruitment and informed consent, clinical assessment, sample collection and storage, and data collection utilizing printed CRFs and EDC systems.

#### The week prior to enrollment (− 1~0 week)

The following procedures will be completed:

1. Written informed consent will be obtained from the participants for the study, and the screening questionnaire will be completed.
2. Office blood pressure and heart rate will be recorded.
3. Previous history, current and prior treatment, and other documented eligibility factors will be recorded.
4. Personal information such as height, weight, and waist circumference will be recorded.
5. Examinations including 24-h ABPM, electrocardiography, routine blood tests, blood biochemical tests, routine urine tests, urine microalbumin, creatinine, and arterial stiffness assessments (PWV, ABI) will be performed.
6. Participant eligibility will be reconfirmed according to the inclusion and exclusion criteria.
7. The baseline case report form (CRF) with printed CRF and EDC system will be completed.
8. Fecal and blood samples will be collected.
9. The appointment for the randomization visit will be made (within a week after screening).

#### Randomization (day 0)

Participants will be randomly assigned to receive the first capsule treatment (day 1), and a dietary history will be collected before treatment.

#### Treatment period (day 1, day 7, day 14)

The eligible subjects will be randomly divided into two arms, in which the “FMT capsule” group and the “placebo capsule” group will receive FMT via capsules or placebo capsules, respectively, three times in the outpatient clinics on day 1, day 7, and day 14 after the trial initiation. At each visit, medication adherence, adverse events, treatment for comorbid diseases, and dietary history will also be assessed and recorded.

#### Follow-up visits (day 7, day 14, day 30, day 60)

1. Office blood pressure and heart rate will be recorded, and on day 30, 24-h ABPM will be performed.
2. Any reasons for discontinuing intervention will be recorded.
3. Adverse events, participant adherence to treatment regimens, and changes in combined medication for comorbid diseases and nonstudy therapy will be recorded.
4. Dietary history will be recorded.
5. Blood and fecal samples will be collected.
6. The follow-up CRFs will be completed with printed CRF and EDC systems.
7. An appointment for the next follow-up visit will be made.

#### Final visit (day 90)

Participants will undergo a comprehensive examination to evaluate the efficacy of FMT for hypertension treatment, including physical examination, blood pressure, heart rate, 24-h ABPM, routine blood tests, routine urine tests, blood biochemical tests, urine microalbuminuria protein, creatinine, electrocardiography, and arterial stiffness assessments (ABIs, PWVs). In addition, dietary history, adverse events, and combined medications for comorbid disease will be recorded, and all CRFs will be reviewed. Additionally, effective colonization and durability of intestinal flora in hypertensive recipients after oral administration of “FMT capsules” or “placebo capsules” and the underlying mechanism of BP change and the fecal microbiota will be analyzed via metagenomic and metabolomic profiling analysis.

### Plans to promote participant retention and complete follow-up {18b}

All enrolled participants will receive a comprehensive health evaluation via a professional physician and will receive FMT capsules or placebo capsules for free. During the average follow-up of 3 months, participants will receive consultation from professionals, medication guidance, and follow-up services for free. To assist in travel costs and other expenses to take part in the study, subsidies will be provided for each participant per visit. In terms of participants refusing to follow the allocated intervention algorithm or experiencing adverse events, follow-up in reference to the evaluation plan will continue. The reason for withdrawal (and degree of withdrawal) will be recorded for all participants who withdraw from the study or who are not thoroughly followed up. If participants withdraw, clinical treatment and other rights and interests of patients will not be affected.

### Data management {19}

Data collection will be performed via the printed CRF, and then data will be entered into the Electronic Data Capture (EDC) system (http://47.107.145.115/fmtEDC/) developed by the National Center for Cardiovascular Diseases, enabling repeated confirmation from the patient care providers and/or investigators from the implementation centers. All staff will be trained and e-mailed with accounts and passwords for using the EDC system with signed meeting summaries. The EDC system will automatically check the data format, rules, and range, which will then be rechecked manually. All printed CRF and examination results will be scanned and sent to the Collaborating Office with two staff independently reconfirming the accuracy of the data. Changes in data are allowed and tracked with reasons in the EDC system. Additionally, a third party is invited to supervise data quality. After completion of the study, the EDC database will turn to a locked state with the blinding audit report generated by the statistician and confirmed by the principal investigator, responsible data manager, and statistical analyst. Data will be kept for 5 years after study completion.

### Confidentiality {27}

In terms of protection of participant confidentiality, personal information of participants, including name, address, contact, and personal ID numbers, will be kept within investigation centers, and access by any third party will be prohibited. Regarding data collection (i.e., metagenomic profiling, metabolomic profiling, clinical information, and examination results), all personal identifiers will be removed with alternatives such as unique screening code and randomization code used.

### Plans for collection, laboratory evaluation, and storage of biological specimens for genetic or molecular analysis in this trial/future use {33}

Except for routine clinical laboratory tests for blood and urine samples (will not be stored) as described above, study blood (10 ml) and fecal (5 g) samples will be collected at baseline and at each visit. Blood samples will be centrifuged immediately with supernatants and blood cells collected separately. These samples will be stored in − 80 °C freezers and delivered to the responsible center via dry ice for further metagenomic and metabolomic profiling analysis. Other experiments, such as specific bacteria isolated from fecal samples, may be developed to address the scientific questions of the study.

## Statistical methods

### Statistical methods for primary and secondary outcomes {20a}

All primary analyses for end points will be based on the intent-to-treat principal, including all randomized patients received at least a capsule, regardless of adherence. For primary endpoint analysis, analysis of covariance adjusting for baseline SBP and center will be performed. In terms of baseline data and secondary outcomes, categorical variables will be described as frequencies (percentages, %), with differences between the arms evaluated through the Cohran-Mantel-Haensel chi-square test or Fisher’s exact test as appropriate, with center effect adjustment. Continuous variables are presented as the mean (standard deviation (SD)) or median (interquartile range (IQR)), with *t* tests or Wilcoxon tests performed as appropriate. Assessment of interventions according to the change in continuous data will be performed using mixed-effect models or the analysis of covariance. All statistical tests of clinical metadata will be conducted using the SAS®9.4 software or the R software (version 4.0.2). A significance level of one-sided *p* < 0.025 will be utilized for primary endpoint analysis, and a significant *p* value for other statistical analyses is defined as a 2-sided *p* < 0.05.

### Interim analyses {21b}

The interim analysis is deemed unnecessary for this study, given follow-up conditions: (1) short-term study period with only 3-month follow-up for each participant and risk of type I error of interim analysis and (2) a relatively small sample size of 120 patients and blinded setting of study design.

### Methods for additional analyses (e.g., subgroup analyses) {20b}

Further sensitivity analysis to assess the outcome consistency will be performed in multiple subgroups, such as according to age, sex, baseline office SBP level, implementation sites, history of diabetes or hyperlipidemia at randomization, and proportion of donor microbiota engraftment.

### Methods in analysis to handle protocol non-adherence and any statistical methods to handle missing data {20c}

A per-protocol analysis will also be performed and will include randomized patients successfully treated and exclude those presenting with major deviations (i.e., violation of core inclusion and/or exclusion criteria, use of forbidden drugs, intervention protocol violation). In terms of missing data handling, the multiple imputation method will be used for the primary endpoint. Additional sensitivity analyses will be performed with the data using the last-observation-carried-forward (LOCF) method and for the complete dataset excluding missing data. For secondary endpoints, missing data will not be handled because of the exploratory nature of the analysis.

The final protocol for the study and statistical analysis will be identified before locking the EDC database, and the *ClinicalTrials* website will be updated. Additionally, the full protocol, deidentified participant-level dataset, and statistical code will be shared along with the publication according to the journal requirements.

### Oversight and monitoring

#### Composition of the coordinating center and trial steering committee {5d}

The overall study was coordinated and overseen by the Collaborating Office and the Quality Control Committee following Good Clinical Practice (GCP) principles with online catch-up meetings every week. Additionally, the web-based EDC system allows for real-time monitoring, and scanned CRFs and examination reports will be rechecked by staff. Each implementation center will also organize a local investigation team including the local principal investigator ensuring study protocol implementation and quality control; trained investigators and/or patient care providers for participant screening, recruitment, follow-up, and adverse event handling; and staff for data collection and storage and specimen collection, processing, and storage.

#### Composition of the data monitoring committee, its role, and reporting structure {21a}

To monitor the trial process and participant safety, the data and safety monitoring committee was established, including experts of hypertension diagnosis, management, biostatistics, and other necessary fields, in reference to the STEP trial [34]; all personnel were completely independent from the funders and any competing interests. The staff of the Collaborating Office and a third party from the Medical Research & Biometrics Center, National Center for Cardiovascular Diseases, Fuwai Hospital Chinese Academy of Medical Sciences, were invited for data monitoring with periodical reports to the data and safety monitoring committee. Data and safety monitoring committee meetings will occur with onsite visits once at each site every 3 months to conduct process evaluation, participant safety monitoring, and recommendation to the trial steering committee in terms of protocol change, process continuation, and others. Of note, decisions are only made by the steering committee.

#### Adverse event reporting and harms {22}

Potential risks for FMT include transmission of currently unknown infectious agents or diseases related to donor gut microbiota, although a profound screening process was performed according to the current consensus. Other previously reported adverse events generally have mild and mainly self-limiting safety concerns (minor adverse events), such as abdominal discomfort, altered bowl habits, bloating, nausea, vomiting, flatulence, transient low-grade fever, and borborygmus, particularly in the FMT route via oral capsules [35]. Adverse events, as one of the secondary outcomes in the study, are identified as any discomfort or clinical symptoms within the study period. In addition to adverse events specifically queried by physicians at each follow-up visit, adverse events are evaluated at onset, for instance, according to the complaints by the participants or observations by the study staff. Beginning with the first intervention, all adverse events will be recorded on the AE page of the CRF and EDC system with details of onset date and time, severity, procedures, correlation, and influence on study interventions, prognosis, and date. The severity of AEs and their correlation with study interventions will be assessed according to the Common Terminology Criteria for Adverse Events (CTCAE Version 4.03, http://evs.nci.nih.gov/ftp1/CTCAE/CTCAE_4.03_2010-06-14_QuickReference_8.5x11.pdf) with a five-grade scale. Severe adverse events (SAEs) are defined as events leading to mortality, need for or prolongation of hospitalization, persistent or severe morbidity, and/or medical emergency determined by investigators in accordance with the GCP. Investigators will deal with and report SAEs to the local ethics committee immediately and to the responsible center and principal investigator within 24 h. Documented AEs will be evaluated by the staff from the Collaborating Office and a third party from the Medical Research & Biometrics Center, National Center for Cardiovascular Diseases, Fuwai Hospital Chinese Academy of Medical Sciences, within 3 days after each visit and reported to the data safety and monitoring committee in 3-month intervals. All adverse events will be reported in the trial publication.

#### Frequency and plans for auditing trial conduct {23}

The study will be periodically audited online and onsite by qualified staff of the Collaborating Office and the Quality Control Committee, with experience from the Strategy of Blood Pressure Intervention in Elderly Hypertensive Patients (STEP) trial [34].

#### Plans for communicating important protocol amendments to relevant parties (e.g., trial participants, ethical committees) {25}

The study protocol will not be changed without written approval from the Ethics Committee of the responsible center. Protocol amendments will also be submitted to the ethics committee of implementation centers, investigators, and participants.

#### Dissemination plans {31a}

All unpublished information and data related to the study are strictly confidential and will be utilized only for this study, as agreed upon by all investigators. The study results will be shared with the participants, published in peer-reviewed journals, and/or presented at academic conferences. Deidentified data will be updated on secure online databases such as the European Nucleotide Archive (ENA, https://www.ebi.ac.uk/ena/submit/sra/) along with publication requirements from the journal. Access to these deidentified data will be required for written permission from the responsible investigation center and only for qualified researchers. To allow for validation and meta-analysis with future studies of FMT for hypertension, we will make our data available to other study groups after reasonable application. Coded samples may be shared with academic collaborators, including metagenomic profiles, metabolomic profiles, and clinical metadata. The sharing will be confirmed with the standard language of the informed consent, and all individual information will be excluded.

## Discussion

A large amount of evidence indicates an absence of microbe involvement in an association or even cause-and-effect relationships of microbial dysbiosis with noncommunicable diseases in the context of different environmental and genetic predispositions [36]. Recent patient- and animal model-based studies have revealed distinct gut microbiota compositions and functions accompanied by host hypertensive or prehypertensive states [5, 7–10]. Of note, our previous study revealed the transfer of an elevated BP phenotype from hypertensive patients to GF rodents, which confirmed a cause-and-effect relationship between the gut microbiota and BP elevation [9]. A hypothesis of gut microbiota intervention was thus proposed with multiple vehicles being investigated to date, such as prebiotics, probiotics, and antibiotics. Prebiotics are dietary components (i.e., fermentable fibers) that interact with microbes, and probiotics are beneficial living microbes, in some cases contributing to the improved gut barrier function and host homeostasis [37]. A recent study suggested that a high-fiber diet and acetate supplementation showed significant BP-lowering and heart-/kidney-protecting effects in DOCA-salt hypertensive models [38]. Additionally, probiotics such as *Lactobacillus* bacteria [39, 40] presented BP-lowering effects in both animal models and human trials, with a potential mechanistic explanation of angiotensin-converting enzyme inhibition [41–43]. However, a meta-analysis of current prebiotics [15] or probiotics [33] indicated modest BP-lowering effects of − 0.9 mmHg (95% CI -2.5 to − 0.6 mmHg) and − 3.56 mmHg (95% CI − 6.46 to − 0.66 mmHg) for SBP reduction, respectively. The latter meta-analysis also revealed a threshold of duration (> 8 weeks) and daily dose (probiotics > 10^11^ colony-forming units) and a recommendation of multiple bacteria prescribed for effective intervention [33]. Additionally, innovative products such as mixed strains of live bacteria belonging to *Lactobacilus* and *Bifidobacteria* are still under trial [44]. Limited evidence of specific microbes causing hypertension prohibits the exploration of microorganism-targeting supplements via prebiotics and/or probiotics or inhibition via antibiotics. In addition, broad-spectrum antibiotics, which are commonly utilized for infectious disease treatment and preintervention of experimental models for microbiome studies, probably disrupt microbial homeostasis with the potential loss of beneficial species or strains and cumulatively increase the future risk of antibiotic-resistant microbes and even pathogenic conditions such as obesity, diabetes, asthma, and inflammatory bowel diseases [45–49]. Our study thus focused on a methodology of healthy gut microbiota restoration (FMT) and has already been performed or investigated in the contexts of multiple infectious diseases and non-communicable diseases. In addition, oral capsules are used to reduce the risk associated with invasive delivery methods such as upper endoscopy, colonoscopy, and nasoenteric tubes [35]. The study is the first to explore the effect of FMT on hypertension with cardiovascular event-associated indicators [32], with office SBP as the primary outcome, and other BP-related indicators, including office DBP and BP indicators from ABPM as the main secondary endpoints. The target population is constrained to patients with grade one hypertension defined according to the “2010 Chinese guidelines for the management of hypertension” [28], in whom nonpharmacological treatment options can be recommended first for those without secondary causes, severe target organ damage, or accompanying diseases. Further explorations of the changes in the recipient microbiome and metabolome via multiomics approaches are planned for an advanced understanding of the role of specific microbes in hypertension. In terms of stability of the microbiota, products interfering with microbiota, such as antibiotics or probiotics, were prohibited during the 3-month study period. Given the recall bias and practical conditions leading to challenging recruitment, the duration of a month for antibiotic or probiotic use prior to recruitment was included in the exclusion criteria. Additionally, a strict multicenter, randomized, blinded, placebo-controlled trial will be performed to ensure the reliability of the study results.

In summary, this is the first study aiming to provide high-quality evidence of a microbiota intervention to treat hypertension via fecal microbiota transplantation as an advancement and clinical translation of our previous research on the role of the gut microbiota in the pathogenesis of hypertension. Further investigations on specific microorganisms or related postbiotics may also be developed from this study via multiple approaches, such as metagenomic sequencing and metabolomic profiling.

## Trial status

This article is in accordance with the study protocol (version 3.3, dated 26 February 2021). Participant recruitment started on 17 March 2021, with 120 participants recruited by 16 September 2021, and is anticipated to finish follow-up visits by December 2021.

## Abbreviations

FMT: Fecal microbiota transplantation
CVD: Cardiovascular disease
BP: Blood pressure
GF: Germ-free
IBD: Inflammatory bowel disease
BMI: Body mass index
ABI: Ankle-branchial index
SBP: Systolic blood pressure
DBP: Diastolic blood pressure
LAACI: Large artery atherosclerotic cerebral infarction
TIA: Transient ischemic attack
PCI: Percutaneous transluminal coronary intervention
CABG: Coronary artery bypass grafting
NYHA: New York Heart Association
ABPM: 24-h ambulatory blood pressure monitoring
AE: Adverse event
ECG: Electronic cardiography
PWV: Pulse wave velocity
ALT: Alanine aminotransferase
AST: Aspartate aminotransferase
K: Potassium
Na: Sodium
Cl: Chloride
BUN: Urea nitrogen
CREA: Creatinine
UA: Uric acid
TC: Total cholesterol
TG: Triglyceride
HDL-C: High-density lipoprotein cholesterol
LDL-C: Low-density lipoprotein cholesterol
GLU: Fasting blood glucose
EDC: Electronic Data Capture
GCP: Good Clinical Practice
ID: Identification
CRF: Case report form
SD: Standard deviation
IQR: Interquartile range
LOCF: Last-observation-carried-forward
CTCAE: Common Terminology Criteria for Adverse Events
STEP: Strategy of Blood Pressure Intervention in Elderly Hypertensive Patients
ENA: European Nucleotide Archive
